# Immunogenicity and effectiveness of an mRNA therapeutic vaccine for HPV-related malignancies

**DOI:** 10.26508/lsa.202302448

**Published:** 2024-03-21

**Authors:** Jing Wang, Ling Ma, Yunfeng Chen, Rui Zhou, Qixin Wang, Tingting Zhang, Dongrong Yi, Qian Liu, Yongxin Zhang, Weiguo Zhang, Yijie Dong, Shan Cen

**Affiliations:** 1 Institute of Medicinal Biotechnology, Chinese Academy of Medical Science, Beijing, China; 2 RinuaGene Biotechnology Co., Ltd., Suzhou, China

## Abstract

Human papillomavirus (HPV) infections account for several human cancers. There is an urgent need to develop therapeutic vaccines for targeting preexisting high-risk HPV (such as HPV 16 and 18) infections and lesions, which are insensitive to preventative vaccines. In this study, we developed a lipid nanoparticle–formulated mRNA-based HPV therapeutic vaccine (mHTV), mHTV-02, targeting the E6/E7 of HPV16 and HPV-18. mHTV-02 dramatically induced antigen-specific cellular immune response and robust memory T-cell immunity in mice, besides significant CD8^+^ T-cell infiltration and cytotoxicity in TC-1 tumors expressing HPV E6/E7, resulting in tumor regression and prolonged survival in mice. Moreover, evaluation of routes of administration found that intramuscular or intratumoral injection of mHTV-02 displayed significant therapeutic effects. In contrast, intravenous delivery of the vaccine barely showed any benefit in reducing tumor size or improving animal survival. These data together support mHTV-02 as a candidate therapeutic mRNA vaccine via specific administration routes for treating malignancies caused by HPV16 or HPV18 infections.

## Abstract (precis)

mHTV-02 functions as a candidate effective therapeutic mRNA-LNP vaccine via intramuscular or intratumoral administration routes for treating malignancies caused by HPV16 or HPV18 infections.

## Introduction

Infection with human papillomavirus (HPV) is estimated to account for 5% of all human cancer cases (1, 2). HPV genome encodes several open-reading frames, including the early (E1, E2, E4, E5, E6, and E7) and late (L1 and L2) proteins. The HPV E6 and E7 oncoproteins interact with and inactivate the tumor suppressors p53 and retinoblastoma protein (pRb), respectively, and drive oncogenic transformation and immortalization. Currently, more than 200 different types of HPV have been identified, which are classified as low risk (LR-HPV) or high risk (HR-HPV) based on their oncological potential. HPV-16, 18, 31, 33, 35, 39, 45, 51, 52, 56, 58, 59, and 68 have been identified as high risk. Persistent infection by HR-HPV is causatively associated with almost all cervical cancers as well as a subset of anal, penile, and head and neck cancers (3, 4). HPV16 and HPV18 are responsible for 50–60% and 10–20% of cervical cancer cases, respectively, and considered as the most prevalent types among the HR-HPVs. A number of prophylactic vaccines have been developed for preventing HPV infection and reducing HPV malignancies, but they could not eliminate established HPV infections and lesions (5, 6, 7, 8). For many individuals with HPV-associated malignancies, such as cervical cancers and head and neck squamous cell carcinomas, the standard treatments include surgery, chemotherapy, and radiotherapy (9, 10), whereas their success rates depend on the disease extent. New treatment, such as effective therapeutic vaccines, is urgently needed against HR-HPV–associated tumors.

The expression of HPV E6 and E7 antigens in transformed cells and their excellent immunogenicity make them ideal targets for therapeutic HPV vaccines. Accordingly, a number of therapeutic vaccine strategies have been studied, and some of them have completed phase III clinical trials in patients such as with high-grade squamous intraepithelial lesions (11, 12, 13, 14). These vaccine strategies include peptide-based, protein-based, nucleic acid–based, live vector–based, and cell-based vaccines targeting these HPV proteins (15, 16, 17, 18, 19, 20, 21, 22), whereas their application are often limited by the moderate immunogenicity for peptide-based vaccines, the need of a complex delivery system with the risk of integration for DNA vaccines, and the high cost for cell-based vaccines.

The recently developed mRNA-based vaccines have formed a promising platform for infectious diseases and cancer immunotherapy (23, 24). Two mRNA vaccines against SARS-CoV-2 have been successfully developed by Moderna and Pfizer/BNT, respectively. BNT113, an RNA-LPX vaccine targeting HPV16 E6 and E7, is in clinical trials for HPV16-related cancers (NCT03418480 and NCT04534205). HPV16 RNA-LPX represents another mRNA vaccine encoding HPV16 E7 and has been shown as an efficient treatment in a mouse model when administered intravenously (25). However, mRNA vaccine simultaneously targeting both HPV16 and HPV18 is still needed, which will address the unmet need for HPV18-infected patients, given that HPV18 subtype exhibits a higher transforming ability than HPV16 and is implicated in rapid-onset cervical cancers (26, 27).

Therefore, we developed a lipid nanoparticle (LNP)–formulated mRNA-based therapy targeting the E6/E7 of both HPV16 and HPV18. The mRNA vaccine, named mHTV-02 hereafter, dramatically induced antigen-specific T-cell immune response in both healthy and TC-1 tumor–bearing mice. Furthermore, mHTV-02 vaccination in animals with established tumors expressing HPV16 E6/E7 led to strong CD8^+^ T-cell infiltration and cytotoxicity in TC-1 tumors, robust memory T-cell immunity, accompanied by tumor regression and improved overall survival in tumor-bearing mice. Intramuscular and several other administration routes are more effective in efficacy than intravenous delivery. These data together suggest that mHTV-02 holds the potential for the development of a therapeutic mRNA vaccine for treating malignancies caused by HPV16 or HPV18.

## Results

### Design of the HPV mRNA-LNP vaccine

**Figure 1.**
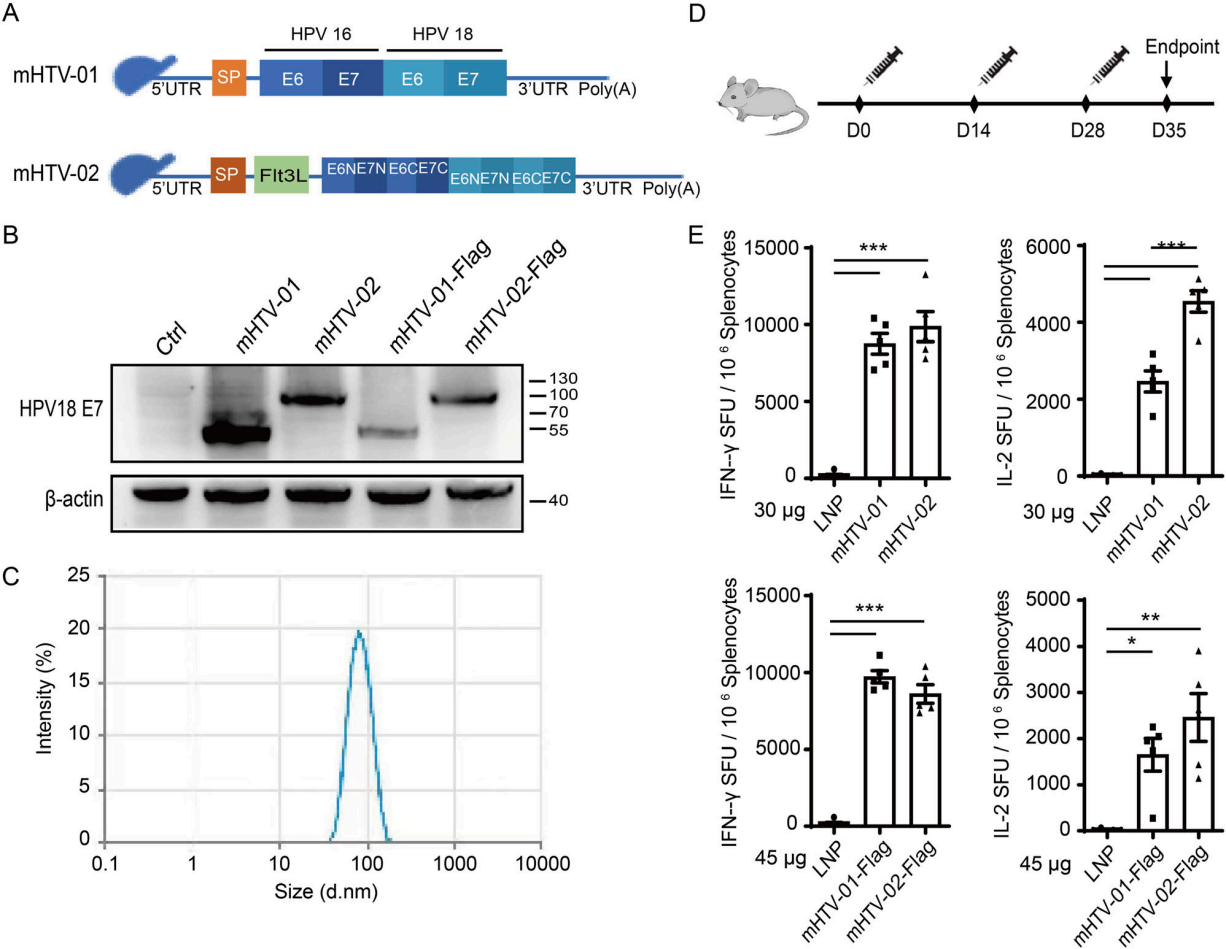
mHTV mRNA-LNP vaccines induced significant T-cell immunity against HPV16/HPV18 E6/E7 antigens in vivo. **(A)** Schematic representation of mHTV-01 and mHTV-02 mRNAs. Signal peptide (SP) (orange) and (Flt3L) (green) are indicated. **(B)** The indicated mRNAs were transfected into HEK293T cells, and the antigen expression was detected by Western blotting using an anti-HPV18 E7 antibody. Actin was used as a loading control. **(C)** Representative of particle size of mRNA-LNP by dynamic light scattering. **(D)** Scheme of vaccination. Mice (n = 5 mice for each group) were vaccinated three times with mHTV-01 or mHTV-02 (30 μg), or FLAG-tagged mHTV-01-Flag or mHTV-02-Flag (45 μg) on days 0, 14, 28. **(E)** Productions of IFN-γ and IL-2 in splenocytes stimulated by the HPV16/18 E6 and E7 were determined by ELISpot assay 7 d after the final vaccination. Mice immunized with empty LNPs were used as negative controls. Data are shown as mean ± SEM and analyzed by one-way ANOVA with multiple comparisons tests (*P ≤ 0.05, **P ≤ 0.01, ***P ≤ 0.001).

The E6 and E7 proteins of HPV16 and HPV18 are promising candidates for developing HPV therapeutic vaccines. Therefore, we chose the four proteins as target antigens and designed two RNA vaccines (mHTV-01 and mHTV-02, respectively) (Fig 1A). mHTV-01 RNA encodes HPV16 and HPV18 E6/E7 in sequence inserted by the endoproteolytic cleavage site, with the IgE leader sequence fused to its N terminus to improve protein secretion. mHTV-02 RNA encodes the reshuffled HPV16 and HPV18 E6/E7 N terminally fused with the tissue plasminogen activator (tPA) signal peptide and the extracellular domain of Fms-like tyrosine kinase-3 ligand (Flt3L) to facilitate antigen presentation. Both RNAs were transfected into HEK293T cells to verify fusion protein expression by Western blotting with an HPV18 E7-specific monoclonal antibody (Fig 1B). We observed specific protein bands with apparent molecular weights of 58 kD for mHTV-01, and 86 kD for mHTV-02 after transfection with the corresponding mRNAs, respectively, consistent with their expected sizes. In addition, after fusing a short sequence encoding the FLAG tag to each mRNA, we detected specific protein bands of expected molecular weights with an anti-FLAG antibody (Fig 1B). Together, these results confirmed the expression of the designed constructs and validated the specificity of the HPV18 E7 antibody.

Next, we evaluated the cellular immune response induced by mHTV-01 and mHTV-02 vaccines in vivo. mRNAs were synthesized by in vitro transcription and formulated into LNPs by T-mixing. Considering that the ionizable lipid SM-102 has been used in Moderna’s COVID-19 vaccine and reported to perform well for intramuscular mRNA delivery (28), we chose SM-102 as the ionizable cationic lipid for our LNP preparations in this study. The encapsulation efficiency of the mRNA-LNP was more than 95% measured by a RiboGreen assay. The representative batches of mRNA-LNP (mHTV-02) had small average particle sizes of 72.82 nm, with a narrow particle dispersion index of 0.034 (Fig 1C). Groups of female C57BL/6 mice were immunized with three doses of mRNA-LNP via intramuscular (i.m.) administration, and mice immunized with empty LNPs were used as negative controls. The last two injections were given at 2 and 4 wk after the first injection, respectively (Fig 1D). Enzyme-linked immunospot (ELISpot) assay was performed to determine the secreted effector cytokines IFN-γ and IL-2 responses to stimulation with a peptide pool from HPV16 and HPV18 E6/E7 proteins. As shown in Fig 1E, both mouse groups immunized with mHTV-01 or mHTV-02 exhibited a marked increase in the secretion of IFN-γ and IL-2 compared with the negative control group. The results indicated that both mHTV-01 and mHTV-02 vaccines induced strong cellular immune responses in mice, with mHTV-02 inducing higher IFN-γ and IL-2 production. Because mHTV-02 appears superior to mHTV-01 in the induction of effector cytokines, we chose mHTV-02 for further investigations.

### Immunogenicity of mHTV-02 mRNA vaccine

**Figure 2.**
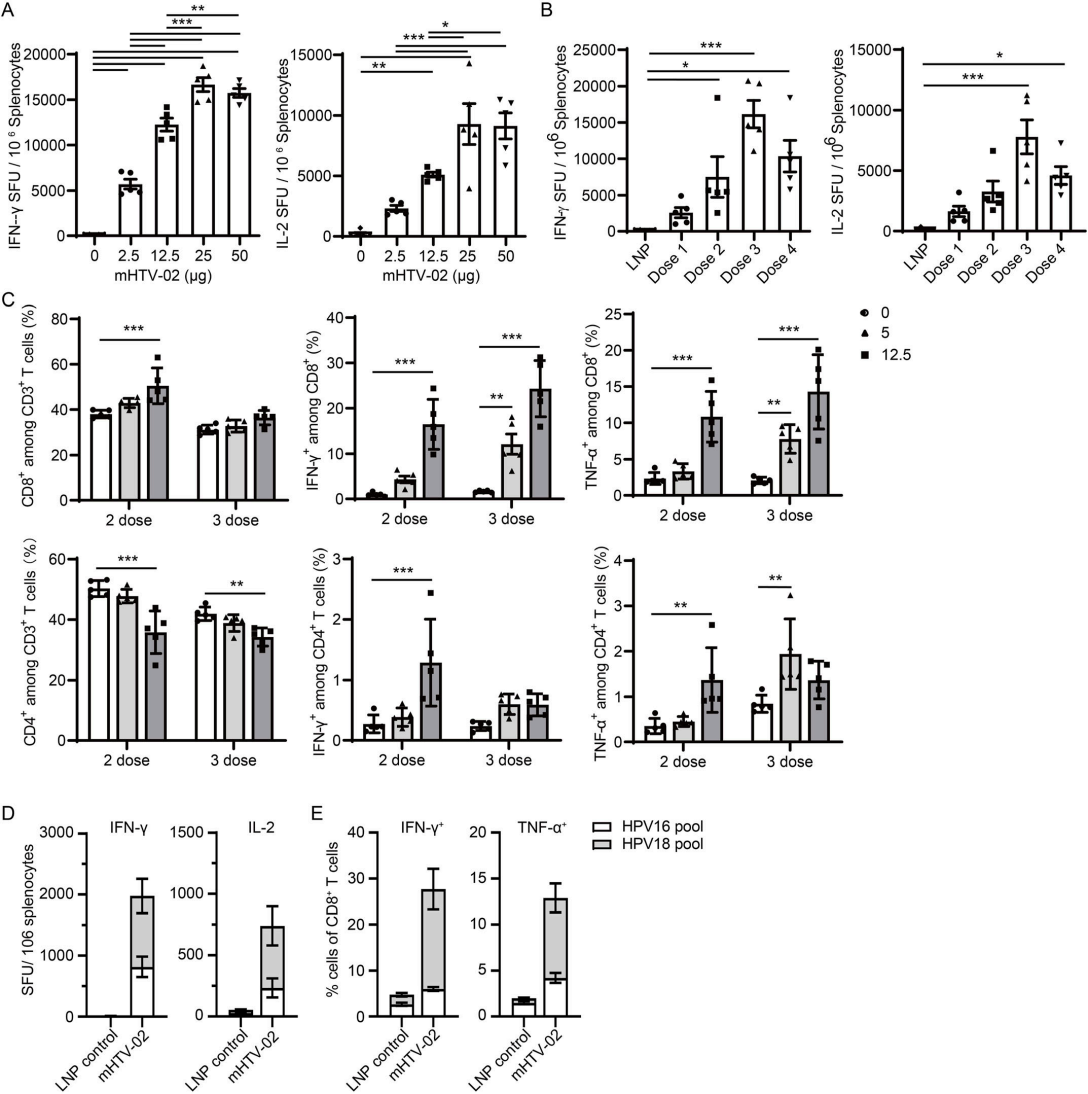
Immunogenicity of the mHTV-02 mRNA vaccine. **(A)** Mice (n = 5 mice for each group) were vaccinated with indicated amounts of mHTV-02 three times at 2-wk intervals. Productions of IFN-γ and IL-2 in splenocytes stimulated by the HPV16/18 E6 and E7 peptides were determined by ELISpot assay 7 d after the final vaccination. Mice immunized with empty LNPs were used as negative controls. **(B)** IFN-γ and IL-2 levels were determined by ELISpot assay in the spleen from mice immunized with one-, two-, three- or four-dose vaccines (n = 5 for each group). **(C)** CD4^+^, CD8^+^, IFN-γ, and TNF-α cytokines were detected by flow cytometry in the spleen from mice vaccinated with indicated amounts of mHTV-02 two or three times at 2-wk intervals (n = 5 for each group). **(D, E)** Mice were vaccinated with mHTV-02 three times at 2-wk intervals for each group (n = 5 mice). Splenocytes were stimulated by the HPV16 pool (HPV16 E6 and E7) or HPV18 pool (HPV18 E6 and E7) 7 d after the final vaccination. Mice immunized with empty LNPs were used as negative controls. **(D)** Productions of IFN-γ and IL-2 in splenocytes were determined by ELISpot assay. **(E)** IFN-γ^+^ CD8^+^ and TNF-α^+^ CD8^+^ cells were detected by flow cytometry. **(A, B, C)** Data are shown as mean ± SEM and analyzed by one-way ANOVA in panels (A, B), or two-way ANOVA with multiple comparisons tests in (C) (*P ≤ 0.05, **P ≤ 0.01, ***P ≤ 0.001).

**Figure S1.**
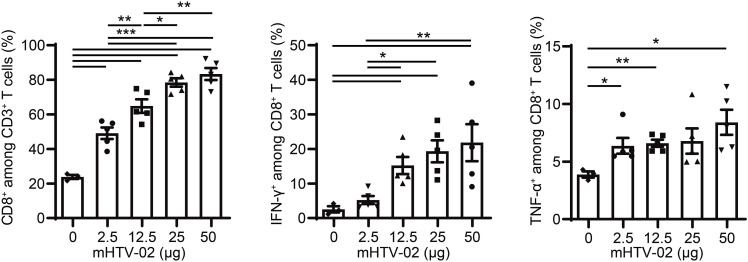
Mice were vaccinated with indicated amounts of mHTV-02 three times at 2-wk intervals for each treatment condition (n = 5). CD8^+^, IFN-γ, and TNF-α cytokines were detected by flow cytometry in the spleen. Data are shown as mean ± SEM and analyzed by one-way ANOVA with multiple comparisons tests (*P ≤ 0.05, **P ≤ 0.01, ***P ≤ 0.001).

To characterize the immunogenicity of mHTV-02 in vivo, groups of five female, 6- to 8-wk-old C57BL/6 mice were immunized via i.m. with 2.5, 12.5, 25, or 50 μg of mHTV-02 vaccine, with empty LNP as the control, on days 0, 14, and 28. Splenocytes were collected 1 wk after the last vaccination, and ELISpot and intracellular cytokine staining (ICS) flow cytometry were performed after stimulation with the E6/E7 peptide pool. ELISpot demonstrated that the vaccine-immunized mice exhibited steeply elevated levels for both IFN-γ and IL-2, reaching a plateau after immunization with 25 μg mHTV-02 (Fig 2A). Similarly, ICS flow cytometry showed that the vaccination with mHTV-02 resulted in a marked increase in E6/E7-specific IFN-γ^+^ and tumor necrosis factor-α (TNF-α)^+^ CD8^+^ T-cell responses (Fig S1).

**Figure S2.**
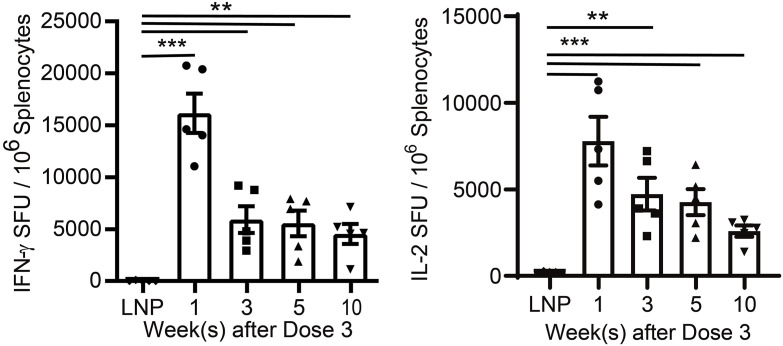
IFN-γ and IL-2 levels were determined by ELISpot assay in the spleen from mHTV-02 immunized mice at indicated weeks after three-dose vaccines (n = 5 mice for each group). Mice immunized with empty LNPs were used as positive controls. Data are shown as mean ± SEM and analyzed by unpaired, two-tailed *t* test between LNP control and mHTV-02 treatment (*P ≤ 0.05, **P ≤ 0.01, ***P ≤ 0.001).

To better develop the vaccination schedule, we compared the cellular immune responses of one- to four-dose injections of the mHTV-02 vaccine. IFN-γ and IL-2 ELISpot analyses at 7 d post-vaccination demonstrated that three immunization doses strongly boosted the E6/E7-specific splenic T-cell responses, compared with the control (Fig 2B). Statistically significant difference between the vaccination and negative control groups was still observed even at 10 wk after the third vaccination (Fig S2), suggesting lasting antigen-specific immune memory induced by the mHTV-02 vaccine.

To identify specific T-cell subsets correlated with mHTV-02 vaccine-induced immune responses, we assessed the ability of CD4^+^ and CD8^+^ T cells to secret effector molecules IFN-γ and TNF-α upon stimulation with E6/E7 peptide pool. As shown in Fig 2C, there was a significant increase in the frequency of antigen-specific IFN-γ^+^ or TNF-α^+^ CD4^+^ and CD8^+^ T cells in the vaccination group, with a higher frequency of CD8^+^ T cells that produced IFN-γ^+^ or TNF-α^+^. The results suggested that the mHTV-02 vaccine strongly activated both CD4^+^ and CD8^+^ T cells specific to HPV antigens.

Because our vaccine was designed to target both HPV16 and HPV18, ELISpot and ICS assays were performed to further determine the cellular immune responses induced by mHTV-02 to stimulations with two peptide pools from HPV16 or HPV18 E6/E7 protein separately. mHTV-02 vaccination induced strong cellular immune responses to both HPV16 and HPV18 E6/E7, indicating potential therapeutic effects on HPV16- and HPV18-driven cancers (Fig 2D and E).

### The therapeutic effect of mHTV-02 mRNA vaccine on the TC-1 tumor model relies on administration route

**Figure 3.**
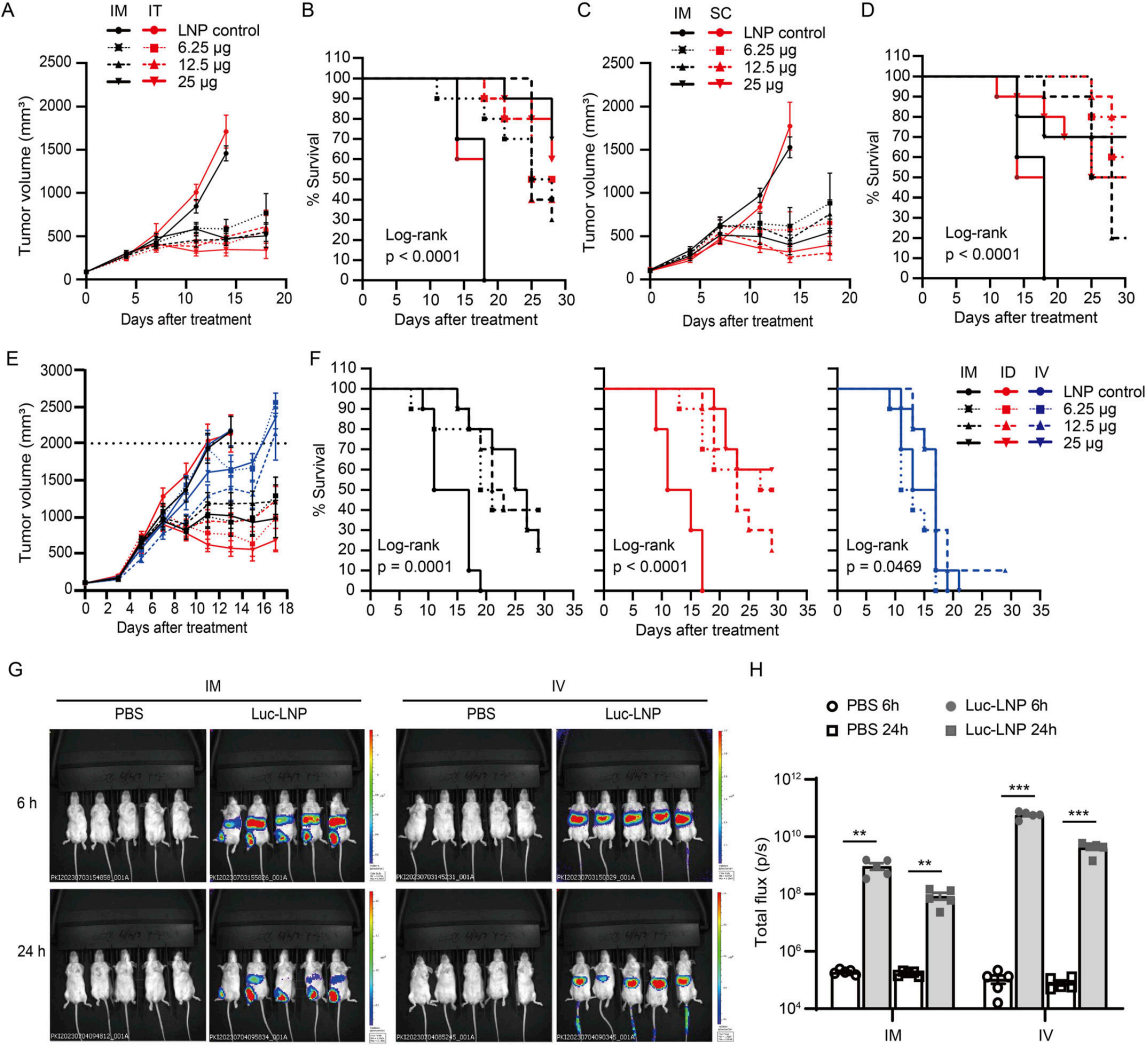
The therapeutic effect of mHTV-02 mRNA vaccine on the TC-1 tumor model. **(A, B, C, D, E, F)** TC-1 tumor–bearing mice (n = 9–10 for each group) were immunized three times at 1-wk intervals with indicated amounts of mHTV-02 or LNP via (A, B) i.m., or i.t. (average tumor size of ∼88 mm^3^ at start of treatment), (C, D) i.m. or s.c. near tumor (average tumor size of ∼105 mm^3^ at start of treatment), and (E, F) i.m., i.d., or i.v. (average tumor size of ∼96 mm^3^ at start of treatment). **(A, B, C, D, E, F)** TC-1 tumor growth and (B, D, F) animal survival were monitored and shown. **(G)** Representative IVIS images of groups of five mice injected with 20 μg mRNA-LNPs via intravenous (i.v.) or 5 μg via intramuscular (i.m.) routes. Mice immunized with PBS were used as negative controls. The scale of luminescence is indicated. **(H)** Quantification of the bioluminescent signal measured at 6 and 24 h after injection. Data are shown as mean ± SEM. **(B, D, F)** Survival curves were analyzed by log-rank (Mantel–Cox) test. **(H)** Statistical significance was analyzed by unpaired, two-tailed *t* test between PBS control and luc-LNP (**P ≤ 0.01, ***P ≤ 0.001).

We next investigated the anti-tumor effects of mHTV-02 vaccination in the HPV16 E6/E7-positive TC-1 tumor model by a dose de-escalation study, as well as assessed the anti-tumor responses using different routes of administration by comparing i.m. versus intra-tumoral (i.t.), i.m. versus subcutaneous (s.c.) near tumor, and i.m. versus intravenous (i.v.) or intradermal (i.d.) injection. C57BL/6 were implanted subcutaneously with 5 × 10^5^ TC-1 tumor cells in the right flank and treated with increasing amounts of mHTV-02 vaccine on days 0, 7, and 14 after tumor volume reached an average of 80–110 mm^3^. The results showed robust tumor growth inhibition for most administration routes except for i.v. (Fig 3A, C, and E) and significantly extended overall survival (Fig 3B, D, and F) at a dose of 6.25 μg of mHTV-02 mRNA-LNP, compared with the control. mHTV-02 administrated via i.t., i.d., or s.c. conferred similar or slightly improved therapeutic efficacy relative to that by i.m. Interestingly, i.v. delivery only mildly inhibited tumor growth with no significant survival benefit. We also observed up to 40% of complete regression of established tumors, especially in the i.t. and i.d. groups. Inferior immunogenicity and therapeutic effect of mHTV-02 associated with i.v. administration is unlikely caused by reduced expression because luciferase mRNA encapsulated by SM-102-based LNP under identical condition via i.v. delivery demonstrated significantly higher enzymatic activity than via i.m. (Fig 3G and H). The results are consistent with the key role of APC activation at or near the i.m. injection site for mHTV-02 mRNA-LNP vaccine–mediated immunogenicity.

**Figure 4.**
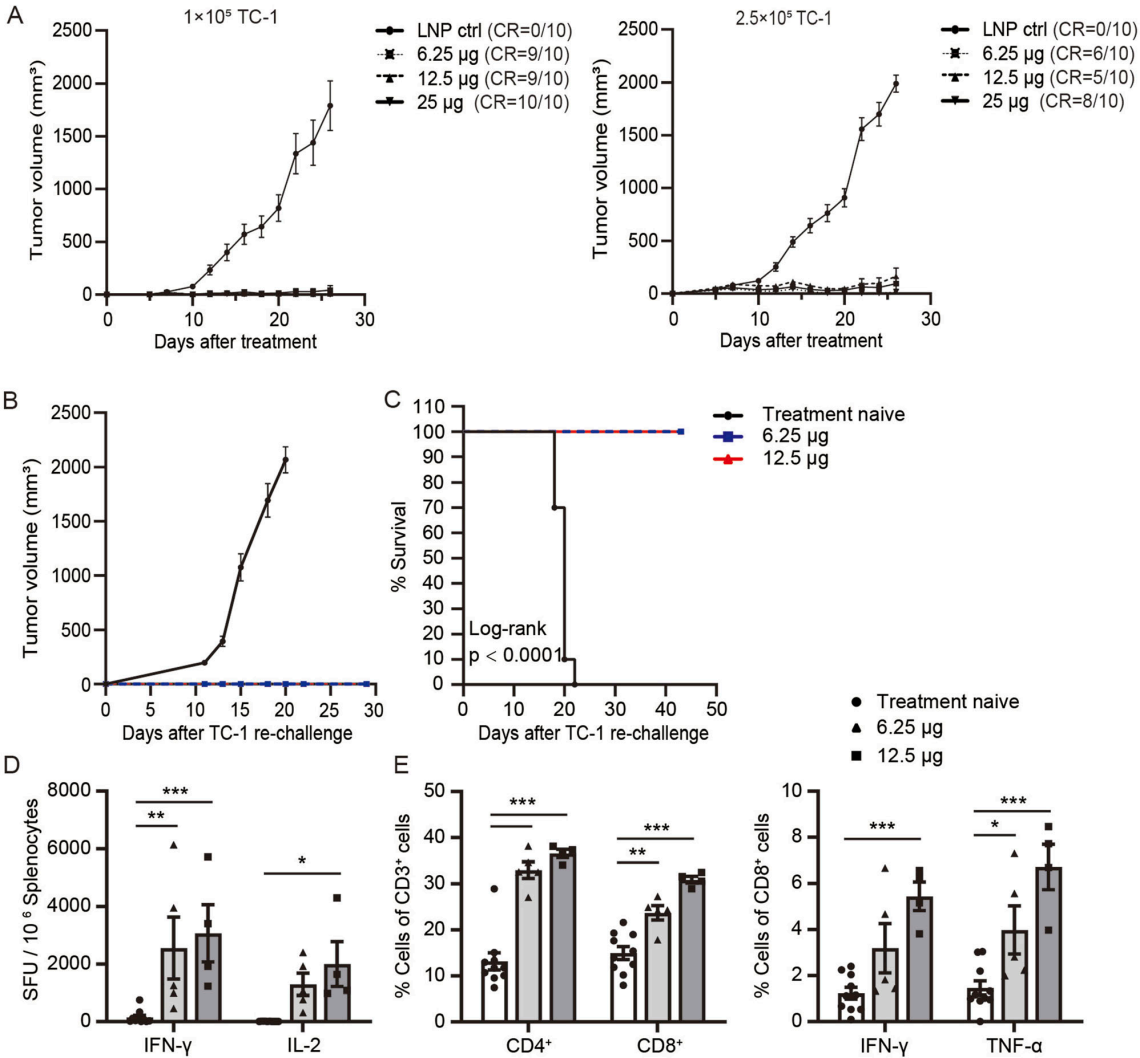
The therapeutic efficacy at an earlier stage of TC-1–challenged mice and long-term anti-tumor memory induced by mHTV-02 mRNA vaccine. **(A)** Mice (n = 10 for each group) were injected with (left) 1 × 10^5^ or (right) 2.5 × 10^5^ TC-1 tumor cells. 3 d later, mice were immunized with indicated amounts of mHTV-02 vaccine three times at 1-wk intervals. TC-1 tumor growth was monitored and shown. **(B, C, D, E)** Mice with complete regression of tumors upon mHTV-02 treatment (6.25 μg and 12.5 μg, n = 5) were rechallenged with TC-1. Treatment-naive mice (n = 10) served as the control group. **(B, C)** Tumor growth and (C) survival were monitored. **(D, E)** Mice were euthanized at the end points. **(D, E)** IFN-γ and IL-2 levels were determined by ELISpot assay, and ((E), left) the proportion of CD4^+^, CD8^+^ T cells, ((E), right) IFN-γ, and TNF-α cytokines in CD8^+^ T cells were measured by flow cytometry in splenocytes stimulated by the HPV16/18 E6 and E7 peptides. Data are shown as mean ± SEM. **(D, E)** Significance was analyzed by two-way ANOVA with multiple comparisons tests (*P ≤ 0.05, **P ≤ 0.01, ***P ≤ 0.001). **(C)** Survival curves were analyzed by log-rank (Mantel–Cox) test.

We further assessed whether mHTV-02 displays better vaccine efficacy in earlier stage of engrafted TC-1 tumor model, compared with that of more advanced established tumors. C57BL/6 mice were injected with 1 × 10^5^ or 2.5 × 10^5^ TC-1 tumor cells. 3 d later, mice were immunized with various amounts of mHTV-02 vaccine three times at 1-wk intervals and tumor volumes were monitored (Table S1). mHTV-02 vaccination with different doses resulted in complete regression in 6–10 of 10 TC-1 tumor–challenged mice, with 100% survival. In contrast, the control LNP-treated mice experienced progressive tumor growth, with 60% (1 × 10^5^ cell inoculation) and 40% (2.5 × 10^5^ cell inoculation) survival 26 d after the first immunization (Fig 4A). These results suggested that earlier mHTV-02 vaccination induced more significant efficacy and prolonged survival compared with treatment at advanced stages of tumor development.

Table S1 The average of tumor size (mm^3^) in all cases of the earlier mHTV-02 vaccination.

To test the long-term anti-tumor memory, surviving mice with complete regression of progressive tumors upon mHTV-02 treatment were rechallenged with the same TC-1 tumor cells in the contralateral dorsal skin 54 d after the last dosage. The tumor growth, survival, and immunological protection against tumor rechallenge were determined. Compared with rapid tumor growth in the normal naïve mice, all animals with a complete response after mHTV-02 treatment rejected TC-1 tumor rechallenge (Fig 4B) and remained tumor free for at least 42 d (Fig 4C), suggesting long-term immunological memory against the E6/E7 antigens. This was further supported by increased production of antigen-specific IFN-γ and IL-2 by ELISpot (Fig 4D) and elevated frequencies of CD4^+^ and CD8^+^ memory T cells, as well as of antigen-specific IFN-γ^+^ or TNF-α^+^ CD8^+^ T cells by flow cytometry in the spleens of mHTV-02–treated mice (Fig 4E). We conclude that the vaccine provided substantial protection against tumor rechallenging.

### Immune cell infiltration induced by mHTV-02 vaccination in mouse tumors

**Figure 5.**
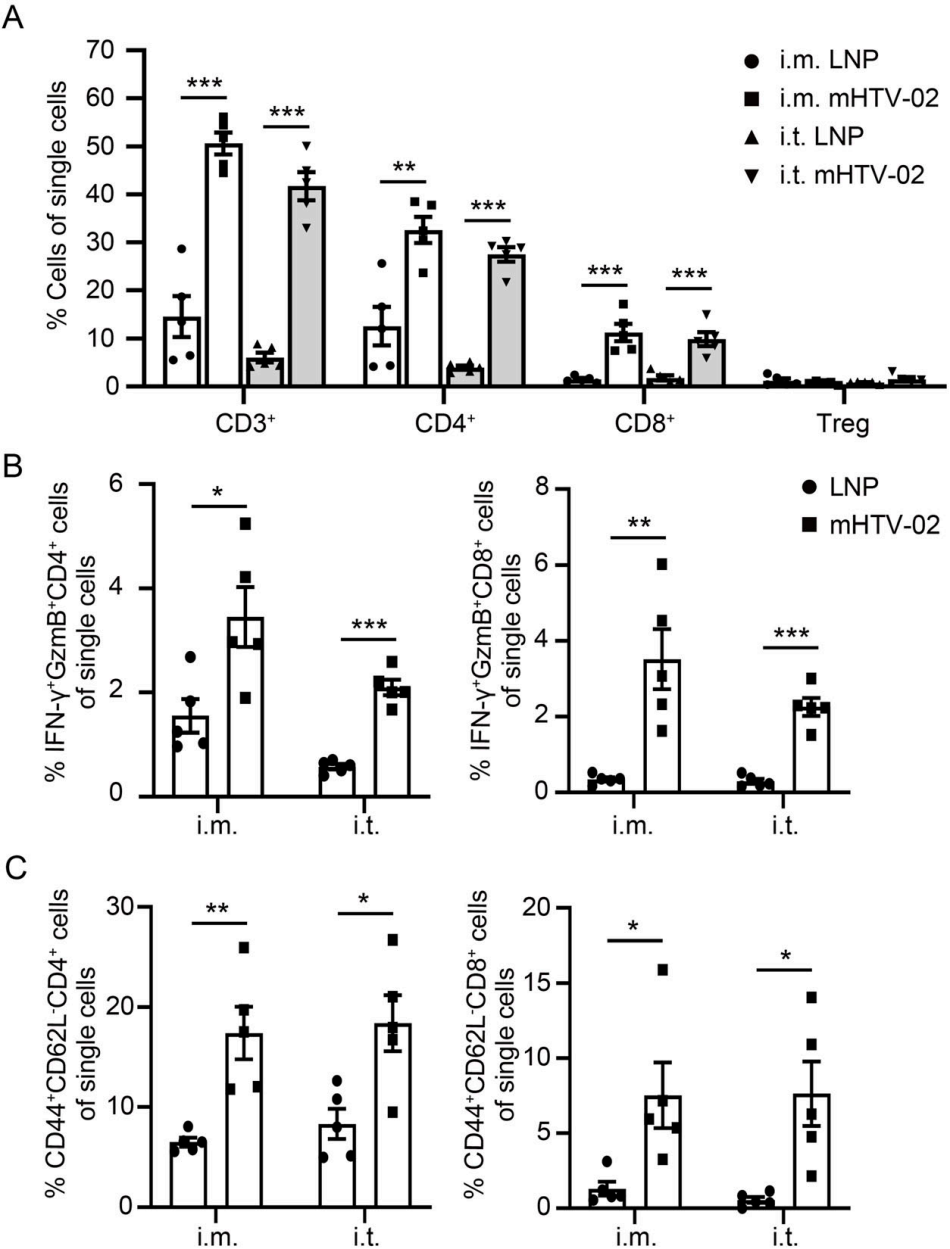
Immune cell infiltration induced by mHTV-02 vaccination in mouse tumors. **(A, B, C)** Mice bearing TC-1 tumors (average size of 97 mm^3^) were immunized twice with mHTV-02 (n = 5 mice for each group). **(A, B, C)** 6 d later, (A) the frequencies of lymphocyte subsets, (B) the frequencies of IFN-γ^+^ GzmB^+^ CD4^+^ T and IFN-γ^+^ GzmB^+^ CD8^+^ T cells, and (C) the frequencies of CD44^+^ CD62L^−^ CD4^+^ T and CD44^+^ CD62L^−^ CD8^+^ T cells in the tumor were measured by flow cytometry. Data are shown as mean ± SEM. Statistical significance was analyzed by unpaired, two-tailed *t* test between LNP control and mHTV-02 treatment.

To understand how the HPV vaccine led to tumor regression, we studied immune cell infiltration in tumors after vaccination. Enhanced migration of antigen-specific T cells to the tumor site is crucial for an effective therapeutic mRNA vaccine. Therefore, we characterized tumor-infiltrated T-cell populations in TC-1 tumor–bearing mice 6 d after the second immunization via i.m. or i.t. The percentages of CD8^+^ and CD4^+^ T cells, memory T cells, and regulatory T cells (Tregs) were examined by flow cytometry of single-cell suspensions of tumors. We observed significantly higher frequencies of CD4^+^ and CD8^+^ T cells in mHTV-02–treated mice than the control group, whereas the frequencies of Tregs were similar between the two groups (Fig 5A). CTLs mediate direct cytotoxic effects on tumor cells upon the releasing of granzyme and perforin, as well as secretion of IFN-γ and TNF-α (29, 30). Tumors of mHTV-02 immunized mice were infiltrated by more multifunctional (IFN-γ^+^ GzmB^+^) CD8^+^ T cells upon ex vivo stimulation with E6/E7 peptide pool in comparison of those from the control group (Fig 5B). Of note, vaccine-treated mice also exhibited threefold and sixfold increases in intra-tumoral effector memory T cells (TEM) in CD4^+^ and CD8^+^ T cells, respectively (Fig 5C). These results indicated that vaccination with mHTV-02 was apt to promote the generation of memory T cells and cytotoxic T-cell infiltration, thus inducing a vigorous CTL-mediated anti-tumor microenvironment.

## Discussion

In this work, we have developed the mHTV-02 mRNA therapeutic vaccine coding for HPV16 and HPV18 E6/E7. We provided evidence supporting that mHTV-02 can induce strong cellular immune responses to viral antigens and anti-tumor effect in mice bearing HPV16-positive TC-1 tumors, resulting in prolonged survival. The TIL analysis suggests therapeutic effects mediated by T-cell immunity. In rechallenging experiment, significant antigen-specific memory T cells in tumors were observed after vaccination, suggesting robust memory T-cell immunity, which is likely responsible for the absence of tumor growth in the TC-1 model.

The aim of this work is to develop a vaccine targeting both HPV16 and HPV18, which is expected to benefit a larger patient population infected with HR-HPV, compared with the mRNA-LPX vaccine against HPV16-related cancers. Although several DNA vaccines were designed for HPV16 and HPV18 (18, 31, 32), their application were limited by weak immune response and risk of genomic integration (33). In consistent with previous studies reporting that mRNA-LNP can be less toxic and more immunogenic than DNA vaccines (34, 35), mRNA-LNP formulated mHTV-02 vaccine elicited more robust cellular immune responses against E6 and E7 antigens than the reported HPV DNA vaccines. Until now, several mRNA-LNP HPV vaccines have been demonstrated to successfully treat HPV-associated tumors in preclinical models. Different from the ionizable lipid SM-102 in mHTV-02, DLin-MC3-DMA or other similar self-developed ionizable lipids were used in gDE7 (36) and HPV mRNA-LNP (37) targeting HPV16 E7. Although SM-102 delivery system was also chosen in the mHTV vaccine reported by Lee et al (38), the lipid composition was SM-102/6,6′-trehalose dioleate/DOPE/butyl lithocholate/DMG-PEG2000 (molar ratio of 25:25:10:38.5:1.5). The different properties in LNP-mediated RNA delivery may contribute to distinct features of these vaccines, such as mRNA transfection efficiency, biodistribution, and administration route. Similar to mHTV-02, low-dose i.m. immunization with gDE7 displayed immunogenicity and therapeutic efficacy in both subcutaneous and orthotopic mouse tumor models. However, i.v. vaccination (37) and s.c. vaccination (38) stimulated more immune response and superior tumor suppression with the other two mRNA-LNP HPV vaccines, respectively.

Flt3L is involved in development and activation of dendritic cells (DCs) and natural killer cells (NK). Previous studies have also shown that Flt3L was associated with accumulation of DCsNK cells and lymphocytes in local tumor tissues (39, 40). Different from mHTV-01 vaccine, the antigen sequence of mHTV-02 vaccine is fused with the extracellular domain of Flt3L at the most N terminus in expectation of facilitating antigen presentation and intratumoral dendritic cell infiltration.

The administration routes for mRNA vaccine are associated with the safety and efficacy of a vaccine in general. mHTV-02 treatment by i.d., i.m., i.t., and s.c. delivery displayed excellent therapeutic efficacy, but not by i.v. delivery. It is surprising that i.v. injection of mHTV-02 barely had any impact on tumor growth inhibition or animal survival, although SM-102-based mNRA-LNP via i.v. delivery method showed much higher luciferase activity in the whole body and in the liver than i.m. This result suggested additional attributes more critical than antigen expression level in the whole body or the liver for SM-102 based LNP vaccine efficacy. It is worth mentioning that intravenous administration of HPV16 RNA-LPX demonstrated durable anti-tumor efficacy (25). A possible explanation for the different results between mHTV-02 mRNA-LNP and HPV16 RNA-LPX could be by other properties of the two formulations (41, 42). SM-102 showed different adjuvant and toxic effects from LPX in a recent study (43). Several factors might contribute to this phenomenon. For example, the abundance of APCs near i.m. injection sites plays a vital role in antigen processing and presentation. It is conceivable that more APCs are stimulated in i.d. and i.m., and i.t. and s.c. delivery, which may facilitate APC presenting antigens to T cells and other immune cells in or near tumors. In addition, since HPV-associated tumors developed in mucosal sites, intranasal route of vaccination has been shown to be effective in inhibiting tumor growth in HPV-associated head and neck tumor, as well as genital tumors (44, 45). Therefore, it is worth to test whether intranasal administration of the mHTV-02 vaccine could induce a strong mucosal immunity and exhibit more effective tumor inhibition than other routes, which needs further investigation.

mHTV-02 vaccination induced significantly stronger T-cell responses and more multifunctional (IFN-γ^+^ GzmB^+^) CD8^+^ T-cell infiltration in the tumor. These results on T-cell responses are correlated with anti-tumor efficacy, suggesting the critical role of CD8^+^ T cells in their anti-tumor efficacy. Although further investigation is still needed, we found that depletion of CD8^+^ T cells, but not CD4^+^ T cells, significantly abrogated anti-tumor activity using an updated version of mHTV-02 (data not shown). In line with our conclusion, other groups showed that anti-tumor effects associated with other mHTVs were abrogated by depletion of CD8^+^ T cells (36, 46). In addition, we observed that intramuscular administration of the vaccine induced intratumoral effector memory T cells (TEM) CD8^+^ T cells in the subcutaneous TC-1 tumors, whereas it is currently unclear whether the CD8^+^ T cells have a phenotype of memory resident T lymphocytes (Trm) that have shown an important anti-tumor role, which is also worthy of further investigation.

In conclusion, our study demonstrated the therapeutic potential of the mHTV-02 mRNA vaccine for HR-HPV–driven cancers and pre-cancerous lesions via robust and long-lasting T-cell immunity. It will be interesting to determine if mHTV-02 can enhance the efficacy of anti–PD-1 immunotherapy in future studies.

## Materials and Methods

### Mice

Female C57BL/6 mice or BALB/c (6–8 wk old) were purchased from Charles River and kept under standard and pathogen-free conditions (25°C, 50 ± 10% humidity, a 12-h dark/light cycle) with free access to food and water. All animal procedures and experiments were approved by the Institutional Animal Care and Use Committee of the Institute of Medicinal Biotechnology of the Chinese Academy of Medical Sciences.

### mRNA synthesis and LNP formulation

pCDNA-mHTV-01 and pCDNA-mHTV-02 plasmids containing codon-optimized HPV16/18 *E6* and *E7* genes with 5′ and -3′ UTRs and a poly(A) tail was synthesized by Genewiz. The linearized plasmids were in vitro transcribed and capped by CleanCap (#ON-134; Hongene Biotech) with N1-methylpseudouridine-5′-triphosphate (m1ΨTP) instead of uridine-5′-triphosphate (UTP). The mRNA was purified and analyzed by microfluidic capillary electrophoresis (Agilent Fragment Analyzer 5200). Purified mRNAs were encapsulated in LNP formulation. Briefly, the lipids were mixed in ethanol containing an ionizable lipid, cholesterol, 1, 2-distearoyl-sn-glycero-3-phosphocholine (DSPC), and PEG-lipid (with molar ratios of 50:38.5:10:1.5). The mRNA-LNP was obtained by combining the lipid mixture with 20 mM citrate buffer (pH 5.0) containing mRNA through a T-mixer. The formulations were tested for particle size and distribution using a Zetasizer Nano ZS instrument (Malvern), RNA concentration, and encapsulation using a RiboGreen assay (#R11490; Invitrogen).

### mRNA transfection and Western blotting

HEK293T cells were transfected with the indicated mRNAs (2 μg) using the Lipofectamine RNAiMAX (Thermo Fisher Scientific) according to the manufacturer’s instructions or with the LNP-formulated RNAs. Cells were harvested 24 h after transfection, and the lysis was analyzed by Western blot as previously described (47). Blotted proteins were detected with the anti-HPV18 E7 antibody (#PA-117384; Thermo Fisher Scientific).

### In vivo immunogenicity

Female C57BL/6 mice (6–8 wk old) were intramuscularly immunized with indicated amounts of HPV vaccine or empty LNP control, respectively. The immunization was performed three times at 2-wk intervals. 1 wk after the third vaccination, spleens were isolated and suspended in 2 ml RPMI-1640 medium containing 10% FBS. Splenocytes were stimulated with peptide pool (2 μg/ml/peptide), or medium only for ELISpot, and with 10 μg/ml brefeldin A (#B8581; Solarbio) for flow cytometry with a 20-h incubation at 37°C. Peptides were synthesized by SBS Genetech. The ELISpot assay was conducted using the Mouse IFN-γ (#2110002; Dakewe) and IL-2 (#3441-4APW-2; Mabtech) ELISPOT kit according to the manufacturer’s protocol. The spots of each mouse were analyzed by the Mabtech IRIS FluoroSpot/ELISpot reader.

### TC-1 tumor treatment experiment

The murine TC-1 tumor cell line, derived from primary lung epithelial cells, was immortalized with HPV16 E6/E7 and transformed with c-Ha-ras oncogene (48), kindly provided by Dr. Mingzhao Zhu (Institute of Biophysics, Chinese Academy of Sciences). For therapeutic tumor experiments, TC-1 cells were harvested during log phase growth. Female C57BL/6 mice were injected with 5 × 10^5^ TC-1 tumor cells subcutaneously. When the tumors reached an average of 80–110 mm^3^, the vaccine was administrated three times at 1-wk intervals unless otherwise specified. Tumor sizes were measured unblinded using an electronic caliper every 2–3 d for calculating tumor volumes using the equation (width × width × length)/2. Animals were euthanized when either mice showed signs of suffering or the average tumor volume exceeded 2,000 mm^3^.

### In vivo bioluminescence imaging

Female BALB/c mice (6–8 wk old, n = 5) were inoculated with 5 μg of the Luc mRNA-LNP via i.m., or 20 μg via i.v. routes, respectively. At 6 h and 24 h post inoculation, animals were injected intraperitoneally (i.p.) with luciferase substrate (Promega). Mice were imaged at 3 min after reaction and fluorescence signals were collected by IVIS Spectrum instrument (PerkinElmer) for 60 s. Bioluminescence values were quantified by measuring photon flux (photons/second).

### Tissue preparation

The tumors were cut into small pieces and digested with Tumor Dissociation Kit (#130-096-730; Miltenyi) at 37°C for 40 min with periodic agitation. An equal volume of RPMI-1640 medium containing 10% FBS was added to stop the enzymatic reaction, and the tumor samples were filtered through the 70-μM cell strainer (#352350; Falcon). The tumor-infiltrating leukocytes were isolated with the Percoll density gradient (#17-0819-01; GE) and then analyzed by flow cytometry.

### Flow cytometry

Flow cytometry analysis was performed on the spleen or tumor single cell suspension. Monoclonal antibodies against CD3, CD8α, CD4, CD44, CD62L, and CD25 for extracellular staining and antibodies against IFN-γ, IL-2, GzmB, and Foxp3 for intracellular staining were purchased from BioLegend. The stimulated single cells were stained for extracellular targets at 4°C for 30 min after viability dyes and blocking using anti-mouse CD16/32 antibody (BioLegend). After washing, the cells were fixed and permeabilized at 4°C for 30 min using the Cytofix/Cytoperm kit according to the manufacturer’s instructions (eBioscience). Intracellular IFN-γ, IL-2, GzmB, and Foxp3 were stained at room temperature for 1 h. After washing, the cells were resuspended in PBS and analyzed for flow cytometry using a CytoFLEX flow cytometer (Beckman Coulter).

### Statistical analysis

Statistical analysis was performed using GraphPad Prism software (version 8.4.2). Data were expressed as means ± SEM. Comparisons between individual and corresponding control group were analyzed by unpaired, two-tailed *t* test. Comparisons of multiple groups were performed using one-way analysis of variance (ANOVA) with Tukey’s multiple comparisons tests. The survival benefit of the tumor-bearing mice was analyzed with the log-rank test (Mantel–Cox), *P* < 0.05 was considered statistically significant.

## Supplementary Material

Reviewer comments
